# Topological properties and resonant tunneling in graphene with Haldane model and extrinsic Rashba effect

**DOI:** 10.1016/j.isci.2025.113354

**Published:** 2025-08-18

**Authors:** Xiao-Long Lü, Jia-En Yang, Hang Xie

**Affiliations:** 1 College of Science, Guangxi University of Science and Technology, Liuzhou, Guangxi 545006, China; 2 College of Physics, Chongqing University, Chongqing 401331, China; 3 Chongqing Key Laboratory for Strongly-Coupled Physics, Chongqing University, Chongqing 401331, China; 4 School of Electronics and IoT, Chongqing Polytechnic University of Electronic Technology, Chongqing 401331, China

**Keywords:** Condensed matter physics, Materials physics

## Abstract

We study the topological phases and resonant tunneling in graphene with the fixed extrinsic Rashba effect. Our study demonstrates that the phase diagram in the space of the Haldane model and ferromagnetic exchange field exhibits a topological phase transition between the Chern numbers C=±2 and C=±4. With the staggered potential, the phase diagram presents the richer topological phases that are characterized by the Chern numbers from C=−3 to C=3. In particular, the Chern number C=0 corresponds to six distinct helical edge states in zigzag graphene nanoribbon, containing the valley edge state and mixed edge state. Additionally, the Chern numbers C=±1 correspond to four valley-polarized edge states. In the junction consisting of different chiral edge states, the transmission manifests many resonant peaks with a transmittance of about 1, originating from the backscattering and different loop currents. Our findings offer insights into the edge states with identical Chern numbers and their resonant tunneling.

## Highlights

• Graphene can exhibit a topological phase transition between the Chern numbers C=−4 and C=4
• Offering a new view to study multiple types of the edge states with identical Chern numbers
• The mechanism of resonant tunneling phenomena of spin-mixed edge state is discovered

## Graphical abstract

**Figure:**
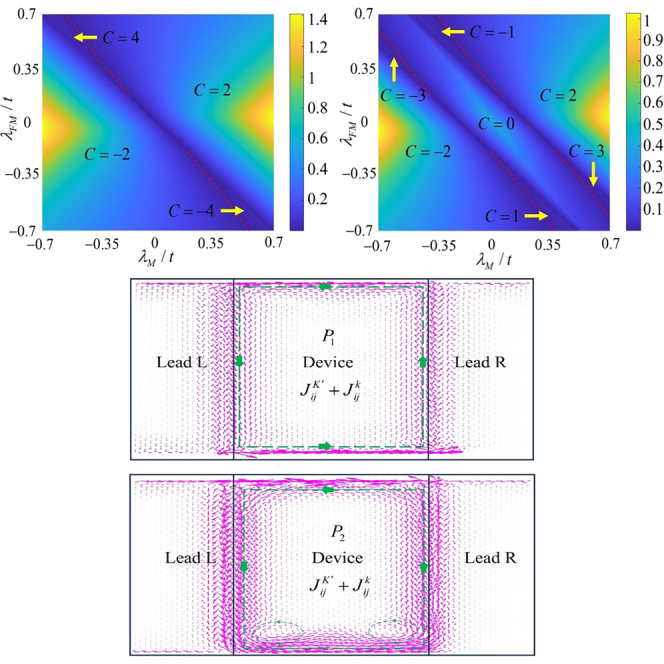

## Introduction

Graphene,^1^ as an extraordinary two-dimensional material, has attracted significant research interest due to its topological effects and potential applications in dissipationless and efficient electronics, spintronics, and valleytronics.^2^^,^^3^^,^^4^^,^^5^^,^^6^^,^^7^^,^^8^^,^^9^^,^^10^ In addition, the charming electronic properties in graphene also contribute significantly to the rapid development of other two-dimensional materials, such as silicene,^11^ germanene,^12^ and stanene.^13^ Recently, the bulk band gaps near two valleys in graphene can be modulated by other external fields^14^^,^^15^^,^^16^^,^^17^^,^^18^^,^^19^^,^^20^ and different types of the spin-orbit coupling (SOC) that include the Rashba SOC,^21^^,^^22^^,^^23^^,^^24^ intrinsic SOC and staggered SOC,^25^^,^^26^^,^^27^^,^^28^^,^^29^^,^^30^ indicating that graphene can host a series of topological phase transitions. Based on the bulk-edge correspondence principle,^31^^,^^32^^,^^33^ these topological phases can give rise to the abundant fascinating edge states in the boundaries of the system, providing a versatile platform for practical applications.

In the presence of the Haldane model,^34^ the chiral edge state associated with the quantum anomalous Hall effect (QAHE)^35^^,^^36^ can be generated, where the edge states counter propagate along opposite boundaries. When intrinsic SOC and external fields are further considered, QAHE can transition into other topological phases, accompanied by corresponding edge-state transformations,^35^^,^^36^^,^^37^^,^^38^^,^^39^ representing the spin-resolved topology. It is demonstrated that the transmission of these nonvalley edge states, in the absence of the Rashba effect, exhibits resonant tunneling, which originates from different loop currents.^5^^,^^20^^,^^40^^,^^41^^,^^42^^,^^43^ In particular, the Rashba SOC plays a crucial role in inducing intriguing topological phases and edge states, representing the spin-mixed topology. For instance, with the Rashba SOC that mixes the spin modes in graphene, the protected pseudohelical edge state can be induced and evolved into other edge states, which can be modulated by staggered intrinsic SOC and external fields.^25^^,^^26^^,^^27^ Furthermore, silicene, as a counterpart of graphene, hosts three types of QAHE in the presence of the Rashba effect,^9^^,^^44^^,^^45^^,^^46^^,^^47^ which are associated with the valley edge states. Actually, the topological phases and edge states observed in silicene can also be realized in graphene, because the generated and modulated methods are theoretically universal in graphene-like materials.

Additionally, the Chern numbers C=±2 in silicene with the Rashba effect correspond to four types of chiral edge states, including the mixed edge states where the valley and nonvalley edge states coexist. The reason why the identical Chern number hosts multiple edge states is that the collapse and stability of the Dirac cones can exist individually and jointly.^45^^,^^47^ It has been known that the Haldane model and Rashba effect each undergo a series of topological phases and edge-state transitions, but their collective effect has been less explored and often overlooked. How this collective effect influences the topology of graphene remains an intriguing topic, which is expected to induce other peculiar and novel topological phases. Meanwhile, a zigzag graphene nanoribbon with this collective effect provides a platform to study multiple types of edge states with identical Chern numbers, and its resonant tunneling.

In this work, we study the phase diagrams in the parameter space of the Haldane model and FM field in graphene with a fixed Rashba effect, and further investigate the corresponding edge states, with a focus on the resonant tunneling of typical chiral edge states. The phase diagram without the staggered potential exhibits four distinct topological phases characterized by the Chern numbers C=±2,±4, where C=±4 that host four pairs of chiral edge states are predicted as the highest Chern numbers in steady monolayer graphene-like materials. Furthermore, the phase diagram with the staggered potential presents richer topological phases, in which the corresponding Chern numbers change from C=−3 to C=3. In particular, these identical Chern numbers can correspond to multiple types of edge states, in which six helical edge states with C=0 and four valley-polarized edge states with C=±1 are remarkably found. We also investigate the resonant tunneling of typical chiral edge states in a two-terminal device, where the propagating channels between the leads and device are opposite. It is demonstrated that the resonant peaks with a transmittance of about 1 are associated with four different types of the energy-dependent scattering mechanisms, which can be attributed to the joint effect of the backscattering and loop current. These results demonstrate that graphene continues to provide new insights into the study of the topological and transport properties.

### Model and methods

We investigate the topological phases and resonant tunneling behaviors in graphene with the Haldane model and extrinsic Rashba SOC, in the presence of staggered potential and ferromagnetic exchange (FM) field, the four-band tight-binding Hamiltonian can be written as^21^^,^^23^^,^^24^^,^^48^

(Equation 1) $$H=-t\sum_{<i,j>\alpha }c_{i\alpha }^{†}c_{j\alpha }+i\lambda_{R}\sum_{<i,j>\alpha \beta }{\left(\sigma \times {\hat{d}}_{ij}\right)}_{\alpha \beta }^{Z}c_{i\alpha }^{†}c_{j\beta }+t_{1}\sum_{\ll i,j\gg \alpha }e^{-iv_{ij}\phi }c_{i\alpha }^{†}c_{j\alpha }+\lambda_{FM}\sum_{i\alpha }c_{i\alpha }^{†}{\left(\sigma_{z}\right)}_{\alpha \alpha }c_{i\alpha }+\lambda_{E}\sum_{i\alpha }u_{i}c_{i\alpha }^{†}c_{i\alpha }+H.c\text{.}\text{,}$$

where $c_{i\alpha }^{†}$ ($c_{i\alpha }$) is the creation (annihilation) operator for site i and spin α, <i,j> (≪i,j≫) represents the summation over all the nearest-neighbor (next-nearest-neighbor) sites. $\sigma =\left(\sigma_{x},\sigma_{y},\sigma_{z}\right)$ are the Pauli matrices for spin, ${\hat{d}}_{ij}=d_{ij}/\left|d_{ij}\right|$ denotes the unit vector from sites j to i. The first term denotes the nearest-neighbor hopping with the strength t=1 taken as the unit of energy. The second term denotes the extrinsic Rashba SOC with the strength $\lambda_{R}=0.1t$ as a fixed parameter, which mixes the states of opposite spins and sublattices, and can be induced in graphene grown on a substrate.^49^^,^^50^ The third term describes the Haldane model, where $t_{1}$ is the next-nearest-neighbor hopping, ϕ is the phase and $v_{ij}=+1$ (−1) stands for the counterclockwise (clockwise) hopping for the sublattices A and B. The Haldane model can be rewritten as other form $i\lambda_{M}/3\sqrt{3}\sum_{\ll i,j\gg \alpha }v_{ij}c_{i\alpha }^{†}c_{j\alpha }$ with the strength $\lambda_{M}=\pm 3\sqrt{3}t_{1}$ as ϕ=±π/2 throughout the paper, which shares the same form with the off-resonant circularly polarized light. The fourth term describes the FM field with the strength $\lambda_{FM}$, which can be generated by experimentally coupling graphene with a ferromagnetic insulator.^51^^,^^52^ The fifth term is the staggered potential with the strength $\lambda_{E}$ and $\mu_{i}=\pm 1$ for the sublattices A and B, indicating that different sublattices possess opposite on-site energies. Additionally, it can be induced by an h-BN substrate in graphene.^53^^,^^54^ H.c. stands for Hermitian conjugate.

**Figure 1:**
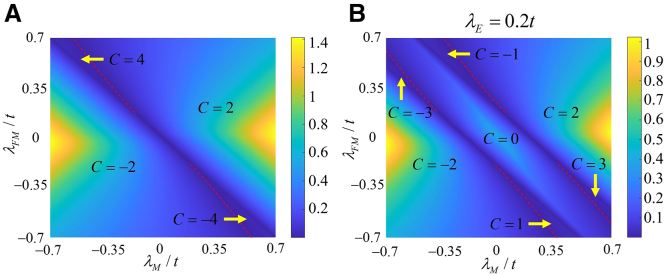
Phase diagrams in the $\left(\lambda_{M},\lambda_{FM}\right)$ plane without and staggered potential (A) $\lambda_{E}=0$. (B) $\lambda_{E}=0.2t$. The colors represent the size of the bulk band gap, and the (red-dotted) lines at the groove denote the phase boundaries between different Chern numbers.

For our system, the Chern number is used to characterize the topological phases, which is written as^24^^,^^55^

(Equation 2) $$C=\frac{1}{2\pi }\sum_{n}\int_{BZ}d^{2}k\;\Omega_{n}\text{,}$$

where n labels all the occupied valence bands and $\Omega_{n}$ is the momentum-space Berry curvature for the *n*th band

(Equation 3) $$\Omega_{n}=-2\text{Im}\sum_{n\neq m}\frac{\langle \psi_{nk}|\partial H\left(k\right)/\partial k_{x}|\psi_{mk}\rangle \langle \psi_{mk}|\partial H\left(k\right)/\partial k_{y}|\psi_{nk}\rangle }{{\left(E_{n}-E_{m}\right)}^{2}}\text{.}$$

m represents both the conduction and valence bands, and a 4×4 matrix H(k) can be obtained by transforming the real-space Hamiltonian in equation 3 into the momentum space. Then, the Block eigenstate $\psi_{nk}$ with eigenenergy $E_{n}$ can be calculated based on H(k). It is worth mentioning that the closing and reopening of the bulk band gap could exhibit the topological phase transition. Therefore, one can first present the diagram of the bulk band gap in the $\left(\lambda_{M},\lambda_{FM}\right)$ plane, where each region is divided by the boundaries, as shown in Figure 1. Then, topological phases can be characterized by the Chern number, which enhances the computational efficiency. According to the bulk-edge correspondence, the Chern number corresponds to abundant topological mixed edge states that the valley and nonvalley edge states coexist in our proposed system. To investigate the resonant tunneling of the mixed edge state, we briefly introduce the transmission coefficient and local bond current for a two-terminal device shown in Figure 6A, based on the wave-function matching technique. The transmission coefficient of electrons injected from lead L to lead R is written as^56^^,^^57^

(Equation 4) $$T_{RL}={\sum_{m}\sum_{n}\left|t_{nm}\right|}^{2}\text{,}$$

where the indices m and n represent the incident mode from lead L and transmitted modes in lead R, respectively. Besides, the normalizing transmission amplitude is expressed as^58^

(Equation 5) $$t_{nm}=\sqrt{\frac{V_{R,n}}{V_{L,m}}}{\tilde{\phi }}_{R,n}^{†}\left(+\right)G_{3}B_{1}^{†}\left\{{\left[F_{1}\left(+\right)\right]}^{-1}-{\left[F_{1}\left(-\right)\right]}^{-1}\right\}\phi_{L,m}\left(+\right)\text{.}$$

The formulas in Equations 4 and 5 could calculate the intervalley and intravalley scatterings as well as the scattering between the valley and nonvalley, which is crucial to investigate different contributions for the resonant tunneling. In particular, the local bond current plays an important role in visualizing the transport path of the topological mixed edge states, reinforcing the understanding of resonant tunneling. With the wave-function technique and the Schrödinger equation, it is described as

(Equation 6) $$J_{ji}^{m}=\frac{i}{\hbar }\left[H_{ji}{\left(\psi_{D,j}^{m}\right)}^{†}\psi_{D,i}^{m}-H_{ij}{\left(\psi_{D,i}^{m}\right)}^{†}\psi_{D,j}^{m}\right]$$

where $J_{ji}^{m}$ denotes the local bond current from sites i to j with the incoming mode m that is incident from lead L.

## Results and discussions

By combining the Chern number and bulk band gap, the phase diagrams with abundant topological phases in the $\left(\lambda_{M},\lambda_{FM}\right)$ plane for monolayer graphene are presented in Figure 1. In Figure 1A, it shows that the monolayer graphene with the Rashba effect can exhibit higher Chern numbers C=±2 and C=±4 in the presence of the Haldane model and FM field. In particular, the higher Chern numbers C=±4 are rarely reported in monolayer graphene-like materials, which could provide four chiral edge states for significantly enhancing the conductance. More interestingly, the phase diagram can be transformed into the one with richer topological phases in Figure 1B, as the staggered potential is considered. One can find that the Chern number varies from C=−3 to C=3. It is worth noting that some Chern numbers in Figure 1 have been investigated in silicene with intrinsic and extrinsic Rashba effects in previous works, but our proposed approach to modulate and generate these phases is fundamentally different. The main difference is that the combination of the extrinsic Rashba effect and Haldane model is studied in monolayer graphene. Additionally, the phase diagram in Figure 1B remains unchanged as the staggered potential is reversed (not shown here). However, the corresponding edge states in a zigzag graphene with identical Chern numbers can undergo the edge-state transition, which is discussed further.

**Figure 2:**
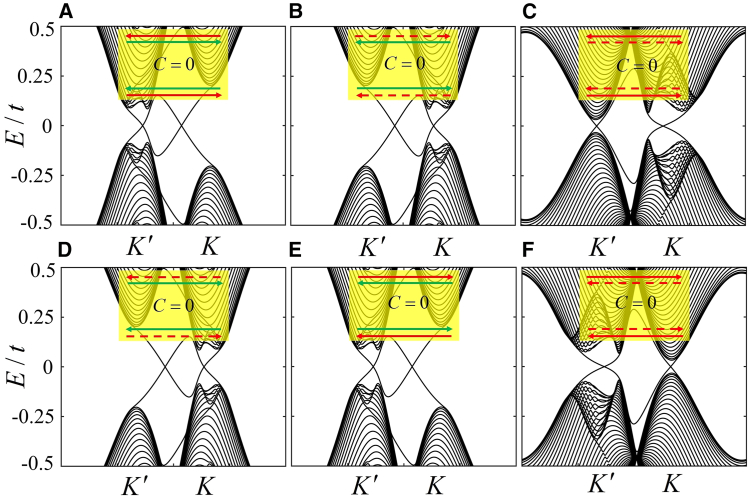
The band structures and corresponding helical edge states in a zigzag graphene nanoribbon with a staggered electric field (A) $\lambda_{M}=0.28t,\lambda_{FM}=-0.28t$ and $\lambda_{E}=0.2t$. (B) $\lambda_{M}=-0.28t,\lambda_{FM}=0.28t$ and $\lambda_{E}=0.2t$. (C) $\lambda_{M}=-0.43t,\lambda_{FM}=0.6t$ and $\lambda_{E}=0.2t$. (D) $\lambda_{M}=0.28t,\lambda_{FM}=-0.28t$ and $\lambda_{E}=-0.2t$. (E) $\lambda_{M}=-0.28t,\lambda_{FM}=0.28t$ and $\lambda_{E}=-0.2t$. (F) $\lambda_{M}=-0.43t,\lambda_{FM}=0.6t$ and $\lambda_{E}=-0.2t$. The red (red-dotted) and green lines in the insets within A-F denote the valley $K^{\prime }$ (K) and nonvalley edge states, respectively. The width of the system is set as $N_{y}=120$ for the number of atoms.

Graphene can exhibit six types of helical edge states for identical Chern number C=0, including the valley edge states and the mixed edge states. In Figure 2A, the band structure corresponds to the mixed edge states, as the gapless bands are located at the valley and nonvalley positions. When the FM field $\lambda_{FM}$ and strength $\lambda_{M}$ of the Haldane model are reversed without changing the staggered potential, the gaps at two valleys in Figure 2A are interconverted; while the $K^{\prime }-\text{valley}$ edge state is accordingly transformed into the K−valley edge state with opposite propagating direction, and the propagating direction of the nonvalley edge state is also opposite, as shown in Figure 2B. When $\lambda_{FM}$ and $\lambda_{M}$ are further strengthened, the bulk gap at the valley $K^{\prime }$ in Figure 2B obviously decreases in Figure 2C, and the nonvalley edge states are switched into the $K^{\prime }-\text{valley}$ edge states with opposite propagating direction, as well as the propagating direction of the valley edge states. As a result, the helical edge state is dominated by valley degrees of freedom. It is worth mentioning that when the staggered potential is reversed, the helical edge states in Figures 2A–2C) can be correspondingly switched into those in Figures 2D–2F) without changing the Chern number, respectively.

**Figure 3:**
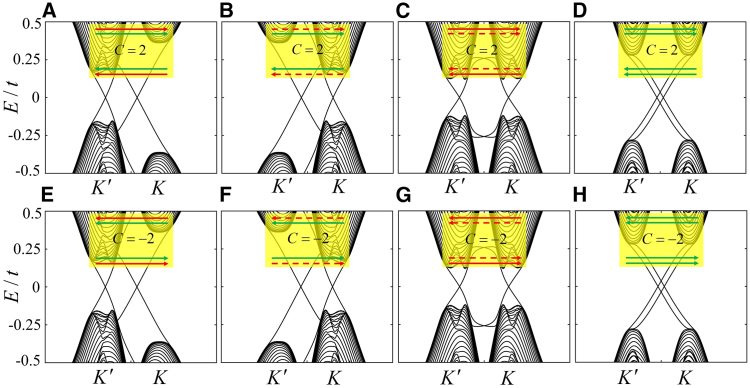
The band structures and eight types of chiral edge states for the Chern numbers C = ±2 The band structures and eight types of chiral edge states for the Chern numbers C=±2, where except the cases in (B) and (C), the rest corresponds to the phase diagram in Figure 1. (A) $\lambda_{M}=0.28t,\lambda_{FM}=0.28t$ and $\lambda_{E}=0.2t$. (B) $\lambda_{M}=0.28t,\lambda_{FM}=0.28t$ and $\lambda_{E}=-0.2t$. (C) $\lambda_{FM}=0.28t$. (D) $\lambda_{M}=0.28t$. (E) $\lambda_{M}=-0.28t,\lambda_{FM}=-0.28t$ and $\lambda_{E}=-0.2t$. (F) $\lambda_{M}=-0.28t,\lambda_{FM}=-0.28t$ and $\lambda_{E}=0.2t$. (G) $\lambda_{FM}=-0.28t$, (h) $\lambda_{M}=-0.28t$. The arrows in the insets within (A–H) and the width of the system are the same as those in Figure 2.

For identical Chern numbers C=±2, eight types of chiral edge states are presented in Figure 3, containing four types of the nonvalley and valley edge states, as well as four types of the mixed edge states. In Figure 3A, the chiral edge states with the Chern number C=2 are shown, dominated by the valley and nonvalley edge states. When the staggered potential is reversed, the bulk band gaps at two valleys in graphene without changing Chern number are interconverted, resulting in that the $K^{\prime }-\text{valley}$ edge state is accordingly transformed into the K−valley edge state with the same propagating direction, as shown in Figure 3B. In the absence of the staggered potential and FM field (Haldane model), the edge states in the inset within Figure 3B can be switched into the valley and nonvalley states in Figures 3C and 3D, respectively. In Figures 3E–3H), we also observe that the Chern number C=−2 corresponds to four types of chiral edge states, where the valley edge states arise from the collapse of the Dirac cones.^45^ Actually, in the presence of intrinsic and extrinsic Rashba effects and off-resonant circularly polarized light, these chiral edge states can be generated in silicene through the application of an FM field and staggered potential. Our results, obtained through alternative methods, indicate that a variety of the edge states for identical Chern numbers generally exist in graphene-like materials with the Rashba effect, which are rarely reported and worth further study of other Chern insulators.

**Figure 4:**
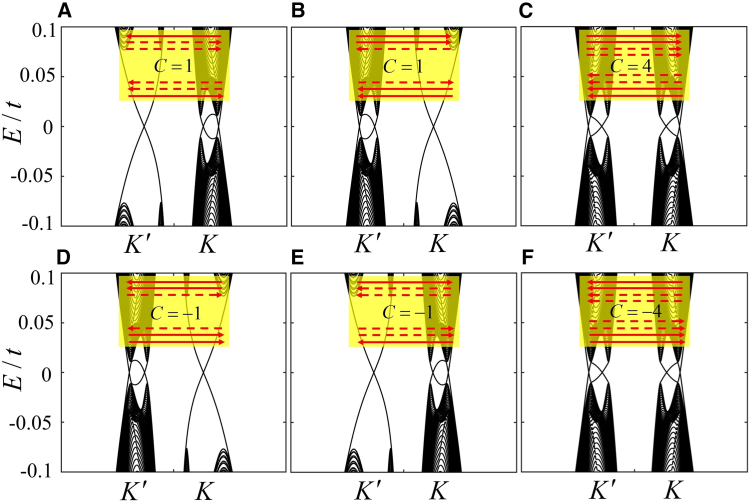
The band structures and corresponding edge states for different Chern numbers (A) $\lambda_{M}=0.39t,\lambda_{FM}=-0.64t$ and $\lambda_{E}=0.2t$. (B) $\lambda_{M}=0.39t,\lambda_{FM}=-0.64t$ and $\lambda_{E}=-0.2t$. (C) $\lambda_{M}=-0.6t,\lambda_{FM}=0.65t$. (D) $\lambda_{M}=-0.39t,\lambda_{FM}=0.64t$ and $\lambda_{E}=0.2t$. (E) $\lambda_{M}=-0.39t,\lambda_{FM}=0.64t$ and $\lambda_{E}=-0.2t$. (F) $\lambda_{M}=0.6t,\lambda_{FM}=-0.65t$. The arrows in the insets within (A–F) are the same as those in Figure 2, the width is set as $N_{y}=480$ representing the number of atoms along the armchair direction. Those in (A), (D), (E), and (F) correspond to the phase diagram in Figure 1, and the cases in (A) and (D) can be transformed into those in (B) and (E), respectively, by reversing the staggered potential.

Here, we discuss the relatively large number of the edge states for the Chern numbers C=±1,±4. Due to the finite-size effect, the emergence of the edge states for these topological phases is presented in wide zigzag graphene. In Figure 4A, three valley edge states with different propagating directions are located at upper and lower boundaries. Based on the bulk-edge correspondence, the Chern number C=1 that is calculated by Equation 2 can also be obtained by directly counting the number of the valley edge state, where the valley-dependent Cherns are $C_{K}=2$ for the valley K and $C_{K^{\prime }}=-1$ for the valley $K^{\prime }$. Consequently, the band structure with C=1 and $C_{V}=C_{K}-C_{K^{\prime }}=3$ exhibits the valley-polarized QAHE. Similarly, the one with C=1 and $C_{V}=-3$ in Figure 4B represents another type of valley-polarized QAHE. In Figures 4D and 4E, there are the other two types of valley-polarized QAHE, where the Chern numbers and valley Chern numbers are $\left(C,C_{V}\right)=\left(-1,-3\right),\left(-1,3\right)$, respectively. Remarkably, the Chern numbers C=±4 are discovered, corresponding to four valley edge states propagating in the same direction in Figures 4C and 4F. So far, C=±4 are predicted as the highest Chern numbers in monolayer graphene-like materials in steady state. To investigate the robustness of the edge states, we choose the chiral edge state in Figure 3A as an example to discuss the effect of the disorder and vacancy on its transmission in the appendix.

**Figure 5:**
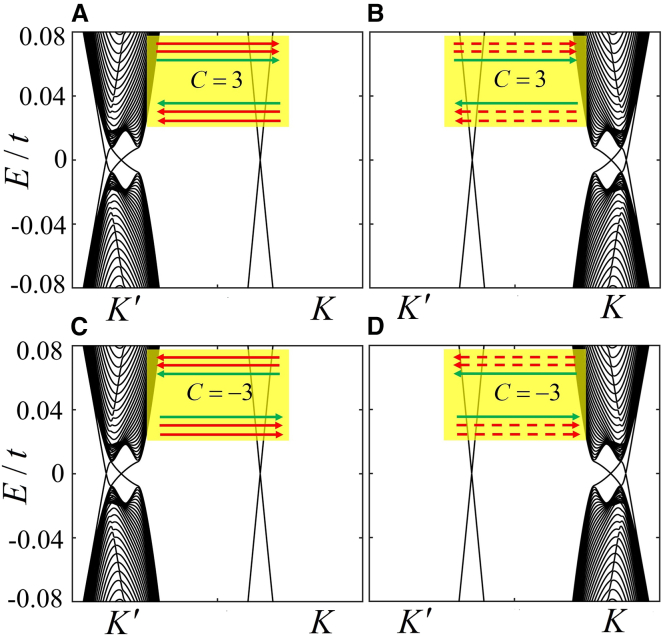
The band structures and corresponding edge states (A) $\lambda_{M}=0.68t,\lambda_{FM}=-0.5t$ and $\lambda_{E}=0.2t$. (B) $\lambda_{M}=0.68t,\lambda_{FM}=-0.5t$ and $\lambda_{E}=-0.2t$. (C) $\lambda_{M}=-0.68t,\lambda_{FM}=0.5t$ and $\lambda_{E}=-0.2t$. (D) $\lambda_{M}=-0.68t,\lambda_{FM}=0.5t$ and $\lambda_{E}=0.2t$. Those in (A) and (D) for the Chern numbers C=±3 in the phase diagram in Figure 1, those in (B) and (C) can arise by reversing the staggered potential in (a) and (d), respectively. The arrows in the insets within (A-D) are the same as those in Figure 3, the width of the system is set as $N_{y}=480$.

By further modulating the strength of $\lambda_{FM}$ and $\lambda_{M}$ based on those in Figure 4, one can obtain two distinct topological phases characterized by the Chern numbers C=±3, and four types of mixed edge states. In Figure 5A, the gapless bands with the Chern number C=3 are located at the valley and nonvalley, resulting in two valley edge states and one nonvalley edge state. When the staggered potential is reversed, the identical Chern number corresponds to the another type of mixed edge state, as shown in Figure 5B. The main difference between these edge states in Figures 5A and 5B is that two $K^{\prime }-\text{valley}$ edge states are converted into two K−valley edge states due to the interchangeability of the bulk gaps modulated by the staggered potential. Moreover, we also observe two types of mixed edge states for the Chern number C=3 in Figures 5C and 5D. Compared with the cases in Figures 5A and 5B, the directions of the mixed edge states are reversed.

**Figure 6:**
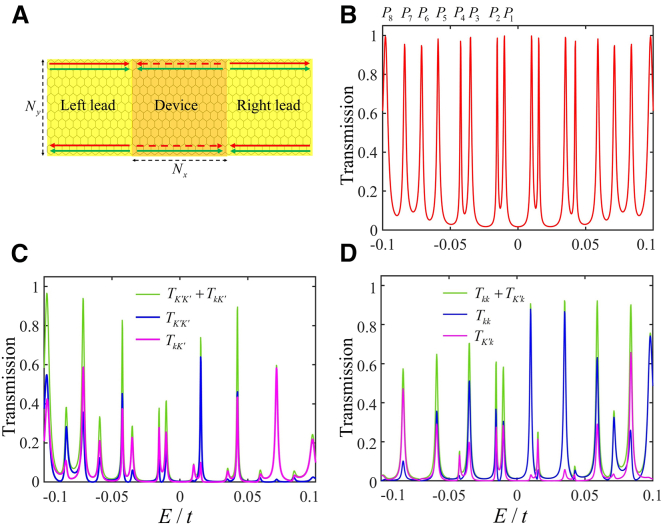
The valley-dependent transmission for a two-terminal device (A) A two-terminal device where two leads are in the same chiral edge state in Figure 3A and the device is in other chiral edge state in Figure 3F, its width is set as $N_{y}=120$ and the length for the device is set as $N_{x}=20$ denoting the number of atoms along zigzag boundary. (B) The total transmission with some peaks denoted by $P_{i}$, arising from all the incoming modes. (C) The transmission coefficients are contributed by incoming mode $K^{\prime }$. (D) The transmission coefficients are from the incoming mode k. The locations of peaks in (C) and (D) are the same as those in (B).

Now we investigate the resonant tunneling of mixed edge states in a two-terminal device by analyzing the transmission coefficient and local bond current. In Figure 6A, the edge-state junction exhibits an opposite propagating direction for two mixed edge states between the leads and device, indicating the interesting and complex transport phenomena. In Figure 6B, the total transmission for the incident modes of the valley $K^{\prime }$ and nonvalley k reveals a series of peaks with a transmittance close to 1, indicating that nearly half of incident current is reflected, due to that two incident modes correspond to the transmittance 2. In Figure 6C, the energy-dependent transmission for the individual incident mode $K^{\prime }$ shows some large and small peaks, as well as the transmission for the incident mode k in Figure 6D. These results indicate that the peaks with a transmittance of about 1 arise from four types of energy-dependent peaks of $T_{K^{\prime }K^{\prime }}$, $T_{kK^{\prime }}$, $T_{kk}$ and $T_{K^{\prime }k}$. Additionally, the general trend, where the number of peaks increases with the device length $N_{x}$ , is not shown here.

**Figure 7:**
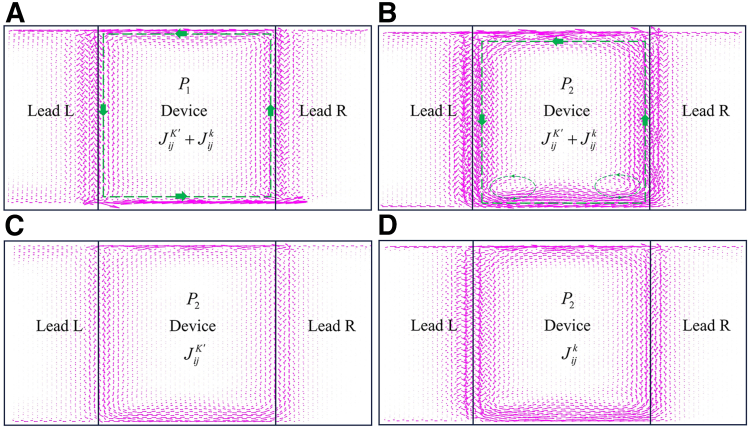
The valley-dependent local bond current for resonant peaks (A) The local bond current with the incoming modes $K^{\prime }$ and k at peak $P_{1}$ corresponds to the transmission coefficient in Figure 6B. (B) The total local bond current with the incoming modes $K^{\prime }$ and k at peak $P_{2}$ in Figure 6B. (C) and (D) are the individual local bond currents with the incoming modes $K^{\prime }$ and k in (B), respectively. The dotted green lines with arrows in (A) and (B) indicate the path of local bond current.

The condition that the propagating directions of the edge states between the leads and device are opposite is expected to generate resonant peaks. For our proposed edge states above, many edge-state junctions can serve as a platform to generate resonant peaks. Here, we select the junction in Figure 6A as a representative example to study the local bond current associated with the resonant peaks. In Figure 7A, the incident current with the modes $K^{\prime }$ and k flowing along the upper boundary in lead L propagates downward along the interface between lead L and device, and then is split into two beams of current, one of which is reflected into lead L and the other propagates into the device. Next, the current in the device propagates along the boundaries of the device multiple times, accompanied by a portion transmitting into lead R. As a result, the backscattering and strong loop current for peak $P_{1}$ arise in Figure 6B. In Figure 7B, the formation process of the loop current for peak $P_{2}$ is the same as the one in Figure 7A, where this stronger loop current consists of two additional small loop currents at the left and right corners. In Figures 7C and 7D, the individual local bond currents $J_{ji}^{K}$ and $J_{ji}^{K}$ are shown, exhibiting the same loop current for the one in Figure 7B. Compared with the incident mode $K^{\prime }$, the contribution of the incident mode k to the total loop current is greater. In addition, the loop currents for peaks $P_{3}-P_{8}$ in Figure 6B belong to those in Figures 7A and 7B.

### Conclusion

In summary, we have demonstrated that graphene with a fixed Rashba effect can exhibit remarkable topological phases and transport properties, which can be modulated by the FM field, staggered potential, and Haldane model. Specifically, in the absence of staggered potential, the phase diagram in the $\left(\lambda_{M},\lambda_{FM}\right)$ plane exhibits that graphene can undergo a series of topological phase transitions between C=±2 and C=±4, where C=±4 are the highest Chern numbers in monolayer graphene-like materials under steady state condition. It is worth noting that the Chern numbers C=±4 correspond to four pairs of chiral edge states with opposite propagating directions, significantly enhancing the conductance. When a staggered potential is applied, the initial phase diagram undergoes a dramatic change, where the Chern numbers vary from C=−3 to C=3. Under these topological phases, there are eight chiral edge states for identical Chern numbers C=±2, six helical edge states for identical Chern number C=0, and four other edge states for identical Chern numbers C=±1 or C=±3. Furthermore, the resonant tunneling of chiral edge states in a two-terminal device is investigated. When the propagating directions between the leads and device are opposite, the total transmission exhibits many resonant peaks arising from a superposition of different scattering mechanisms, which originates from the collective effect of the backscattering and two types of the loop current. These results enhance the understanding of the edge states with identical topological phases, and the resonant tunneling of the edge states.

### Limitations of the study

We theoretical investigated the rich topological phases, multiple types of edge states with identical Chern number, and resonant transport. Although the theories behind these interesting results are highly reliable and widely applied, the realization of related experiments for our predicted results has always been a challenge.

## Resource availability

### Lead contact

Further information and requests for resources should be directed to and will be fulfilled by the lead contact, Xiao-Long Lü (physicslxl@163.com).

### Materials availability

This study did not generate new unique material.

### Data and code availability

• All data reported in this paper will be shared by the lead contact upon request.
• This article does not report original code.
• Any additional information required to reanalyze the data reported in this article is available from the lead contact upon request.

## Acknowledgments

This work is supported by the 10.13039/501100001809National Natural Science Foundation of China (grant no. 12304058), Guangxi Science and Technology Base and Talent Project (grant no. 2022AC21077), National Natural Science of Guangxi Province (grant no. 2024GXNSFBA010229), and the Foundation of 10.13039/501100015920Guangxi University of Science and Technology (grant no. 21Z52).

## Author contributions

X.-L.L.: writing-original draft, investigation, funding acquisition and data curation. J.-E.Y. and H.X.: software and visualization, writing-review and editing. All authors have read and agreed to the published version of the article.

## Declaration of interests

The authors declare no competing interests.

## STAR★Methods

### Key resources table

**Table:** 

REAGENT or RESOURCE	SOURCE	IDENTIFIER
**Software and algorithms**		

MATLAB	MathWorks	https://www.mathworks.com/products/matlab.html

### Method details

The band structure and bulk band gap are calculated by the Schrödinger Equation in the momentum space for zigzag graphene nanoribbon and bulk graphene, respectively. The topological phase diagram is characterized and calculated by the Chern number. The transmission coefficient and corresponding local bond current are calculated by the wave-function matching technique, as well as the effects of disorder and vacancy on the transport properties. All calculated results are obtained in MATLAB R2022a.

## Figures and Tables

**Figure 8:**
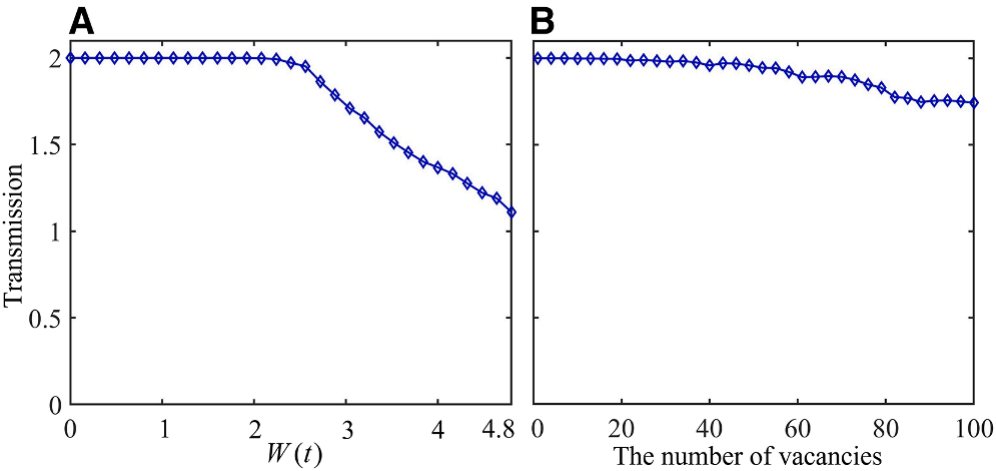
Transmissions for a two-terminal device where two leads and scattering region are all in the chiral edge state (A) Transmission as a function of the disorder strength. (B) Transmission as a function of the vacancy. The energy for the transmissions is set as E=−0.01t, the external fields for the system are set as $\lambda_{M}=0.28t$,$\lambda_{\mathrm{FM}}=0.28t$ and $\lambda_{E}=0.2t$, and the length and width of the scattering region are set as $N_{x}=15$ and $N_{y}=80$, respectively.

## Appendix. The effects of disorder and vacancy on chiral edge state

Here, we discuss the effects of the disorder and vacancy on the edge states. Without loss of generality, the chiral edge state in Figure 3A is chosen as an example in a two-terminal device consisting of two leads and the scattering region. The Anderson on-site disorder energy is considered in the scattering region, where the disorder is randomly distributed within the range of [−W/2,W/2] with the disorder strength W. The vacancy is regarded as an on-site energy with very large strength 2000t to prohibit electrons from hopping to the location of vacancies. In addition, the random disorder and vacancy configurations are both averaged over 200 times to calculate the transmission. In Figure 8A, it exhibits that the quantized transmission is almost not affected by the disorder strength in the range of W≤2.24t, indicating that the chiral edge state is robust against the disorder. However, when the disorder strength is enhanced, the transmission dramatically decreases due to the backscattering from the disorder. Moreover, the transmittance 2 originates from two propagating channels that are provided by the chiral edge states. In Figure 8B, one can find that the quantized transmission is almost steady when the number of vacancies is less than 40. Then, the transmission gently decreases with a large number of vacancies. These results demonstrate that the chiral edge state is moderately robust against the disorder and vacancy.
